# Influence of Renders Surface Structure and Color Properties in the Context of the TLS Accuracy

**DOI:** 10.3390/s25196219

**Published:** 2025-10-08

**Authors:** Andrzej Kwinta, Agnieszka Malec, Izabela Piech, Robert Gradka

**Affiliations:** 1 Department of Land Surveying, University of Agriculture in Krakow, 30120 Krakow, Poland; agnieszka.malec@student.urk.edu.pl; 2 Department of Agricultural Surveying, Cadastre and Photogrammetry, University of Agriculture in Krakow, 30120 Krakow, Poland; izabela.piech@urk.edu.pl; 3 Department of Geodesy and Geoinformatics, Wrocław University of Science and Technology, 50421 Wroclaw, Poland; robert.gradka@pwr.edu.pl

**Keywords:** terrestrial laser scanning (TLS), laboratory studies of point clouds, color analysis, types of decorative renders

## Abstract

**Highlights:**

This paper presents the results of laboratory studies on the impact of the type, color, and brightness of render on the accuracy of measurements performed using the terrestrial laser scanning (TLS). Analyses were conducted for decorative renders of the “Roughcast” and “Scratched” types, with average brightness ranging from 143 to 243. It was found that the standard deviation of the mean distance for “Scratched” renders is approximately 26% higher than for “Roughcast” renders. The results indicate increased dispersion of measurement results as render brightness decreases. Within the analyzed range, it was observed that the blue color has the least impact on the TLS measurement results.

**What are the main findings?**

- Scratched-type render causes a greater standard deviation in TLS measurements than the Roughcast-type one.
- As the brightness of the renders decreases, the dispersion of the statistical parameters of the TLS measurements increases.

**What is the implication of the main finding?**

- Depending on the render structure of the measured object, different TLS measurement accuracies are obtained.
- The darker the render color on the object, the greater the dispersion of the TLS measurement results.

**Abstract:**

This paper presents the results of laboratory research regarding the influence of the structure and color of decorative renders on the accuracy of measurements conducted using Leica ScanStation P40 terrestrial laser scanning (TLS). The study examined whether and how differences in render structure and color (brightness) affect the quality of data acquired via TLS. The color and brightness measurements of the test fields were performed using a flatbed scanner. The RGB color and luminance analysis of the test fields were conducted using the software “ImageJ” version 1.54g. The measurements were conducted for light-colored renders (average brightness from 143 to 243). The research found no clear relationship established between the type and color of render and the accuracy of laser scanning. The results indicate increased measurement dispersion with decreasing render brightness. It was found that the standard deviation of distance measurements for Scratched-type renders is approximately 26% higher than for Roughcast-type render.

## 1. Introduction

Laser scanning technology, including terrestrial laser scanning (TLS), has been considered one of the most significant achievements in surveying over the past decade. The data acquired through this technology provides information that has revolutionized the field of 3D modeling. This measurement technology encompasses a wide range of applications, such as

- Geodesy and engineering (e.g., precise measurements of terrain and engineering structures; creation of three-dimensional infrastructure models; spatial planning and construction process organization) [1,2,3,4,5,6,7];
- Architecture and conservation works (e.g., digitization of objects and creation of digital twins; monitoring of structural deformations; creation or reconstruction of geometric documentation) [8,9,10,11,12,13,14,15,16];
- Forestry and ecology (e.g., creation of 3D forest models, including tree height measurement, crown structure, and biomass distribution; monitoring changes in forest ecosystems; studies on the deforestation or on the impact of climate change) [17,18,19,20,21,22];
- Archeology (e.g., scanning of archeological objects and creation of precise excavation site maps; non-destructive, contactless examination of delicate archeological artifacts) [23,24,25,26];
- Safety and security (e.g., mapping disaster sites; documenting accident scenes; inspecting critical infrastructure) [27,28,29,30,31,32].

The key objective of our study is to investigate the accuracy of measurements made with the use of laser scanning technology. This issue is critical due to the fact that abovementioned technology has been extensively used to acquire 3D data. In our research, the main question was whether the structure and color of render affect the accuracy of surface scanning. Prior studies and publications regarding scanner testing have been lacking laboratory research on a large group of real reflective surfaces (renders) conducted under controlled and constant laboratory conditions.

TLS technology is characterized by high efficiency and precision, enabling the visualization of both surface shape and process kinematics. It provides data that can be used by specialists from various scientific disciplines to interpret ongoing phenomena [1]. Terrestrial laser scanning (TLS) is one of the most accurate methods for surface analysis, allowing for the assessment of surface roughness, structure, and both micro- and macro-textures [33,34]. Due to precise 3D measurements, TLS enables the evaluation of surface irregularities (for example, roughness, defects, and deformations). With the use of TLS, roughness can be analyzed through several key aspects. The first one is the distribution and density of the point cloud. The greater the number of points on a given area, the more accurate the analysis of surface irregularities is, which allows for the precise representation of its structure [35]. Another factor is the intensity of the reflected signal, which can reveal differences in light reflection from various surface fragments [36]. These variations may indicate local texture differences and structural variability of the examined material. Laser beam scattering also plays a significant role. On rough surfaces, light reflects in multiple directions, affecting measurements and potentially increasing their variability [37]. Considering these three aspects allows for a comprehensive analysis of surface irregularities and its characteristics by using TLS technology [38,39]. The analysis of signal reflection intensity in TLS technology is based on measuring the intensity of the laser beam reflected from different surfaces [40]. This enables the identification of materials with different properties, as various structures and chemical compositions influence how light is reflected. Rough surfaces typically cause irregular scattering of the laser beam, which results in variability in signal intensity [40]. Depending on the degree of material roughness and its characteristics, the reflected pulses may have varying values, which provide valuable information during structural analysis of the examined surface [41]. The use of statistical models in surface roughness analysis relies on mathematical processing of data obtained from TLS technology. One of the key parameters used to assess irregularities is the standard deviation of point heights in the cloud. This value allows for a quantitative determination of topographic variability, which is crucial when analyzing diverse surfaces [42]. Additionally, advanced algorithms enable the analysis of surface variability at different scales, allowing for precise determination of both micro- and macro-textures of the studied area. This enables us to obtain a comprehensive picture of the surface structure and its properties, which is applicable in many fields such as geodesy, engineering, and environmental analysis. The accuracy of height and shape measurements in TLS technology may be limited in the case of very rough surfaces, where artifacts or increased measurement uncertainty may occur. Irregularities can cause local disturbances in the laser beam reflection, which results in errors in interpreting the height of individual points in the cloud. Another important factor is laser signal dispersion, which involves the scattering of light in various directions due to contact with an uneven surface structure [40]. This phenomenon can lead to point cloud blurring, making it difficult to accurately reproduce the examined surface. Additionally, the variability in reflection intensity poses a challenge when classifying materials with different properties. Diverse signal reflection can make it difficult to clearly distinguish materials [42,43], especially when they have similar optical characteristics, which affects the accuracy of data analysis and its interpretation. In paper [39], the authors proposed the use of TLS technology to study cones ranging from 5 to 8 m in height made of washed gravel in three different grain size ranges (0–2 mm, 8–16 mm, 32–60 mm). The study employed two modern photogrammetric methods commonly used in the 3D measurements: terrestrial laser scanning (TLS) and the Structure-from-Motion (SfM) technique based on digital images acquired using an unmanned aerial vehicle (UAV). A comparative analysis was conducted to evaluate the potential of the TLS and the UAV methods in measuring the angle of repose (their strengths and weaknesses), and a simplified measurement version was proposed. Both methods yielded very similar results, with measurement uncertainty ranging from 0.4° to 0.7°.

The accuracy of terrestrial laser scanning (TLS) depends on various factors, including surface properties and scanning settings. Different studies have emphasized that the accuracy of TLS is influenced by several factors, including the physical properties of the scanned surfaces, scanning angles, instrument specifications, and configuration settings. Gordon and colleagues [44] demonstrated that TLS can significantly exceed its nominal point precision when measuring deformation. While the typical single-point precision for medium-to-long-range scanners ranges from ±2 mm to ±25 mm, dense point cloud data allows for much greater theoretical surface precision. Their experiments showed that TLS systems could detect vertical deflections with an accuracy six to twelve times better than their specified single-point precision. In one instance, a scanner with ±6 mm point precision achieved a deformation measurement accuracy of ±0.5 mm (RMS), illustrating the potential of TLS in high-precision applications when properly employed. Mala and Al-shrafany [45] focused on how different materials and angles of incidence affect the accuracy of TLS point clouds. Their study found that smooth surfaces such as glass and steel are more sensitive to changes in angle, producing greater measurement errors compared to rough surfaces. For example, at a 0° angle of incidence, the tachymeter target reflected data roughly 20 cm closer to the scanner compared to other materials, while at 75°, the difference was reduced to about 2 mm. Additionally, smooth materials showed higher RMSE values, with glass reaching up to 4 cm at a 45° angle, whereas rough materials generally had RMSEs under 1 cm. These findings underscore the importance of considering both surface reflectivity and roughness when conducting high-accuracy TLS surveys. Brown and Hugenholtz [46] examined the effects of scan resolution and survey configuration on TLS-derived measurements of land surface roughness. They highlighted that survey parameters such as scanner height, distance to the object, and the number of scanning positions have a substantial impact on data accuracy, especially at the centimeter scale. Incorrect configuration choices can lead to distorted representations of surface micro-topography, particularly in applications requiring precise morphological detail. Mills and Fotopoulos [47] addressed the impact of instrument noise and measurement scale on geological surface roughness assessments using TLS. They observed that rangefinder noise can lead to an overestimation of roughness by up to 5%, whereas mismatches between the size of surface features (asperities) and the effective measurement radius may result in underestimation by as much as 20%. The study recommends calibration protocols tailored to specific TLS instruments and target surfaces, as these systematic biases can significantly affect results in geomorphological and geotechnical applications. Overall, these studies collectively highlight that while TLS is a powerful tool for non-contact surface measurement, its effectiveness depends heavily on careful consideration of instrument characteristics, environmental factors, and surface properties. High-accuracy outcomes require not only high-quality hardware but also optimized scanning strategies and calibration tailored to the specific nature of the survey task.

## 2. Materials for Research

**Figure 1:**
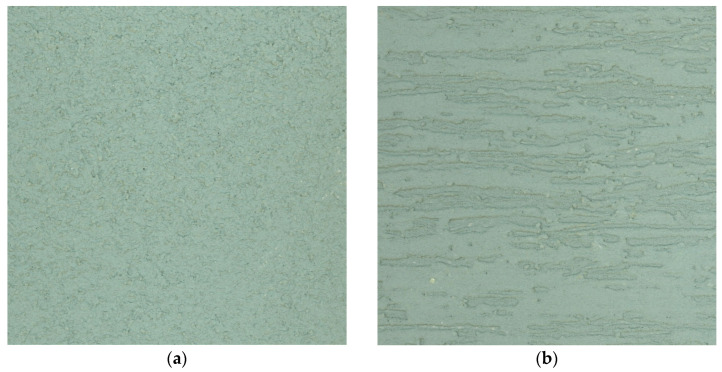
Example structure of decorative render: (**a**) Roughcast render; (**b**) Scratched render.

When examining various construction objects using TLS scanners, it is important to note that these objects are often covered with different finishing materials. Among the many available options, one of the most commonly used solutions is decorative render. These renders can be made from various materials and are characterized by a wide range of colors and textures. In Poland, the most popular renders are made from silicate or silicone, colored with acrylic pigments. The structure of renders can also vary, both in terms of grain size (typically ranging from 1.5 to 3 mm) and application technique. One of the decorative render types, commonly known among renderers as “Roughcast”, is applied to the wall using circular motions or by spaying (Figure 1a). The second most frequently used decorative render is the one with a texture resembling scratched tunnels in wood (Figure 1b). This effect is achieved by applying the render with linear strokes, where the larger grains of the material create the characteristic “Scratched” traces.

In order to conduct the experiment, 83 panels were collected, each panel made of hardboard (HDF) measuring 0.60 × 0.40 m and coated with two types of decorative render in various colors. The panels were sourced from a home improvement store after a display change; therefore, those were in various technical conditions (cracks, damage, splashes, dampness). Half of each panel was covered with Roughcast-type decorative render (Figure 1a), and the other half with Scratched-type render. Unfortunately, some panels were unsuitable for the experiment, and some colors were duplicated. Ultimately, 52 distinct panels were selected, yielding 104 test fields with varying surface structures and colors. Unfortunately, for the study, we could only use the available panels with brightness levels ranging from 143 to 243. For future research, we plan to acquire new panels with a wider range of brightness.

## 3. Course of the Laboratory Experiment

To determine whether different render structures and colors affect the TLS measurements, it was essential to ensure consistent and repeatable measurement conditions. Studies involving instruments and reflective surface parameters are best conducted in laboratory settings [48,49,50,51,52].

Laboratory tests using laser scanners generally focus on two aspects: the accuracy of the instruments and the influence of the reflective surface on measurement precision. Kersten and Lindstaedt [49] tested five different scanners on the same laboratory base. Their findings confirmed the correctness of the data provided in the manufacturers’ technical specifications. Yaman and Yilmaz [50] investigated the impact of surface color on measurement accuracy. From a distance of 35 m, they scanned a 2.1 × 2.8 m test area covered with smooth film in four different colors (white, red, blue, and green). They achieved the best results with the white film. Voegtle, Schwab, and Landes [42] used a Trimble GX scanner from a distance of 14 m to test various reflective materials, including different colored and gray-scale papers, various wood surfaces, and metal plates. Their analysis concluded that the best scanning results (high precision) are obtained by scanning bright, non-specular surfaces at night. Bauer and Woschnitz [51] conducted tests on the Leica RTC360 scanner in a metrology laboratory using a 30 m comparator, with a comparison to interferometric measurements. While the results were satisfactory (showing smaller deviations than stated by the manufacturer), significant systematic factors were identified.

**Figure 2:**
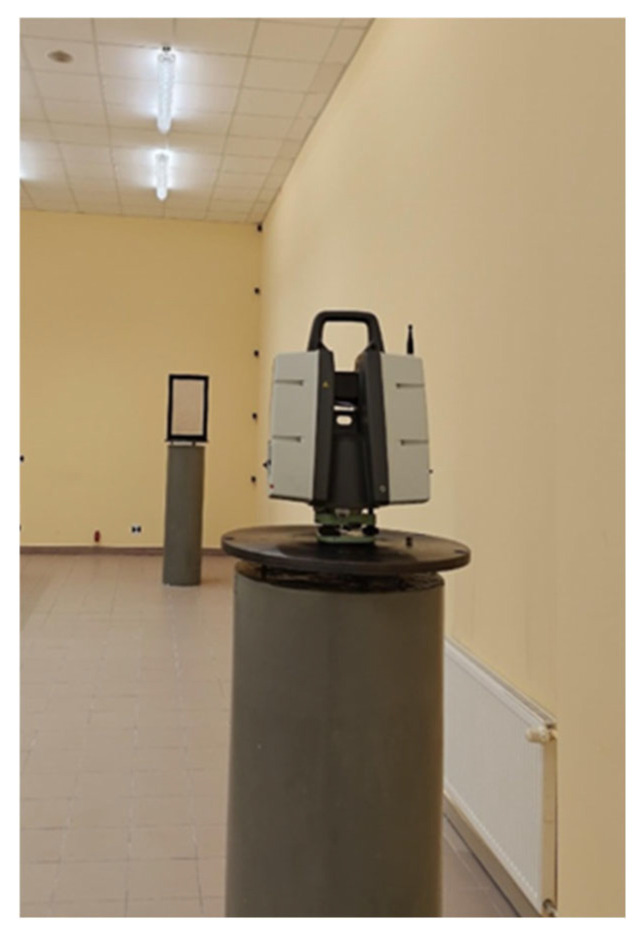
Research station–TLS and a panel with renders mounted in a holder.

The University of Agriculture in Kraków houses a metrology laboratory [53] with dimensions of 37.5 m in length, 6.8 m in width, and 4.2 m in height. The laboratory, located in the basement, is well-insulated from its surroundings. In the laboratory, the climatic and lighting conditions are constant for short periods of time due to the large volume of the laboratory. The measurements were carried out over 8 h. The average temperature during the measurement was 20.6 degrees Celsius (outside the building 26.0 C). Pressure was 1007.8 hPa, and humidity was 66%. During the measurement, the renders were illuminated with the average intensity of 270 Lux (measured with a luxmeter). Among other features, it includes measurement pillars that are 1.42 m high and 0.46 m in diameter. Each pillar is equipped with plates designed for forced centering of geodetic instruments [54], which were used during the measurements to position both the TLS scanner and the test panels. In Figure 2, there is a photograph of the equipment setup during the measurement process.

**Figure 3:**
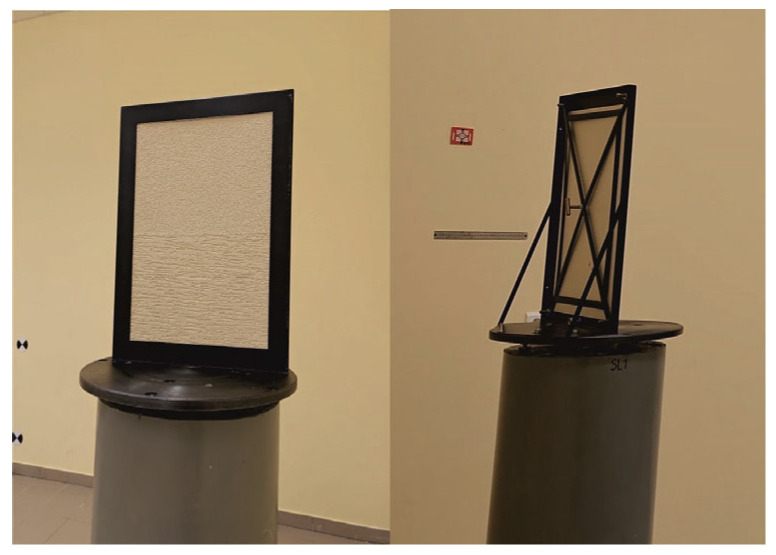
Measurement frame for positioning the panel during scanning.

The Leica P40 laser scanner was mounted on a centering plate using a special mounting bracket [55]. Test panels were placed on a second post approximately 8 m away. To ensure measurement repeatability during tests with different control panels, a customized measurement frame was constructed, in which the panels were mounted for the duration of the measurement (Figure 3).

The measurement frame was made of steel and powder-coated in black. The internal dimensions of the frame were 0.58 × 0.38 m. During measurement, the frame was bolted to the centering plate. The test panel was inserted into the frame from the back so that it fit tightly. It was then secured from behind using a special clamp. In order to ensure repeatable panel positioning during measurements, the clamp was fastened to the frame with four screws (one in each corner) using a torque wrench.

The Leica ScanStation P40 scanner, introduced to the market in 2015, is known for its high measurement precision. Compared to previous Leica devices, it features a more powerful laser that emits more photons. One of the scanner’s main advantages is its ability to effectively measure dark and reflective surfaces. During scanning, the HDR mode can be used, which significantly reduces the time required to generate final products from the acquired point cloud. The Leica P40 enables precise measurements at a rate of up to 1,000,000 points per second over distances of up to 270 m. Its angular range is 360° horizontally and 270° vertically. This scanner can be used under harsh environmental conditions (e.g., in underground mining). Its construction is resistant to water and dust. The instrument operates in temperatures ranging from −20 °C to +50 °C [56].

Terrestrial laser scanning (TLS) enables the acquisition of highly accurate measurements, which allow us, among other things, to create precise three-dimensional models of terrain or objects. TLS scanners can also collect large amounts of data in a relatively short period of time, which significantly speeds up data acquisition in comparison to traditional surveying methods. The TLS can be performed from a distance, eliminating the need for direct contact with the scanned area. Despite its many advantages, TLS effectiveness can be limited by weather conditions such as rain, fog, or intense sunlight, which significantly affect measurement quality. Laser scanners may struggle to accurately measure surfaces with very low or very high reflectivity. Water bodies also pose challenges, as the laser can reflect off its surface, introducing noise into the collected data [57,58].

**Figure 4:**
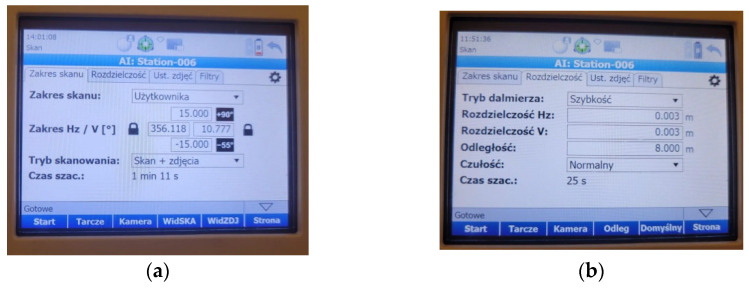
P40 scanner settings during measurement: (**a**) scanning range and mode; and (**b**) configured scanning resolution parameters.

The object was measured using a single scanner station. Before starting the measurement, the instrument had to be properly leveled and configured with basic settings such as vertical and horizontal scanning ranges, vertical and horizontal resolutions of 0.003 m (Figure 4b), a scanning mode, and a distance to the object of 8.000 m. The device was positioned to scan the entire surface of the test panel mounted in the fixed frame. The scanner was operated in “scan + photo” mode, meaning that in addition to the point cloud, photographs were also captured at the station, allowing for visualization of the panels in their original colors. A key feature of the scanner was the ability to quickly define the scanning range in the horizontal plane. Limiting the scanning range to the size of the frame allowed for the acquisition of data solely related to the scanned panels. This reduced both the measurement time and the time required for data processing. The scanner settings are shown in Figure 4.

In order to precisely determine the colors of individual test panels without the influence of lighting conditions on the measurement, a flatbed scanner (for documents) with its own lighting was used. After the TLS measurement, each panel was scanned using a high-quality Plustek OpticPro A320 flatbed scanner. This is an A3-format scanner with high scanning resolution and accurate color reproduction. As a result of this scanning process, 104 uncompressed image files in TIFF (Tagged Image File Format) were obtained at a resolution of 600 dpi. The image dimensions were 7200 × 10,200 pixels.

The measurement results obtained in this way were processed for the purpose of conducting detailed analyses.

## 4. Measurement Data

The initial computational process (processing measurement data for analysis) was divided into two independent stages. In the first stage, point clouds for individual test fields on the control panels were generated based on the TLS measurement data. In the second stage, a color analysis of the panels was conducted.

The direct output from the laser scanning included point clouds in the .bin format and images in the compressed “.jpg” format. The first step involved importing the point clouds into the Leica Cyclone 360 software [59]. In order to do this, a local database had to be created. The next step in project development was to identify elements within the point clouds and to remove fragments unrelated to the analyzed panel. Due to the use of a narrowed scanning range during the measurement, the point clouds were already appropriately limited, which facilitated the elimination of noise and unnecessary elements. The processing of point clouds obtained from the test panel measurements was conducted using Leica Cyclone REGISTER (CORE), an advanced platform dedicated to spatial data analysis [54]. This software offers extensive functionality for modeling, analysis, visualization, and presentation of point clouds. The terrestrial laser scanning data was imported with full geometric accuracy and dataset completeness, while minimizing automation in the initial processing steps. This approach allowed for manual control over key stages of data registration and interpretation. By avoiding filtering and subsampling, the actual measurement environment was accurately recreated [51]. High precision in the point cloud properties was achieved by applying resolution and accuracy parameters at the level of 0.001 m. The processing system configuration was adapted to handle large volumes of data, ensuring both data quality and operational efficiency.

As a result of processing the measurement point clouds, 104 text files in xyz format were obtained (each column represents coordinates in the scanner’s local coordinate system). Each file contains 4489 measurement points arranged in a measurement grid with approximately 3 mm spacing. The grid dimensions are 0.2 × 0.2 m.

The color and brightness analysis of the test fields was conducted for a rectangle measuring 8400 × 6300 pixels. ImageJ software, version 1.54g, was used for image analysis. Within the analyzed rectangle, average RGB color values (*R*—red, *G*—green, *B*—blue) were determined along with the random dispersion values for each color, as well as the medians for each random dispersion. Additionally, the mean brightness of the points was calculated according to the following formula [60,61]:

(1) $$V=\left(R+G+B\right)/3,$$

**Figure 5:**
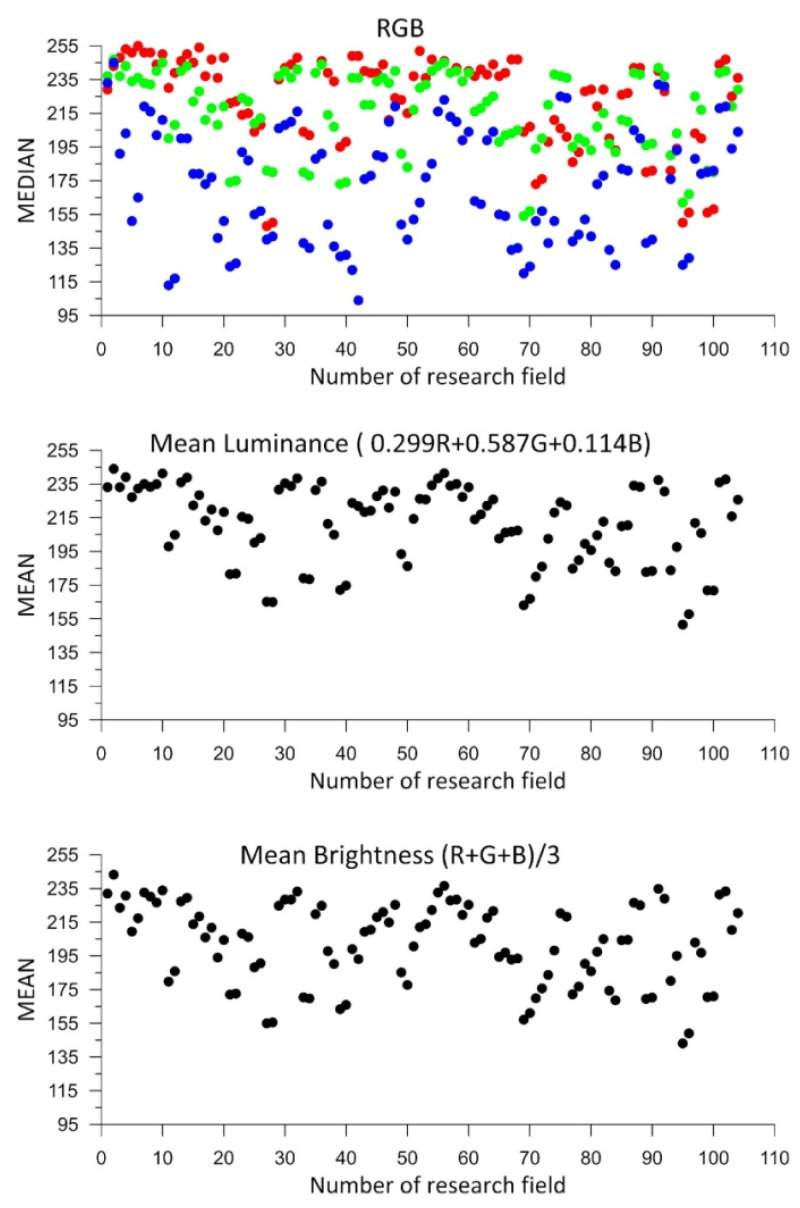
Results of the color analysis of the test panels.

Additionally, the mean luminance value of the pixels was calculated, which is related to the natural perception of colors by humans [61,62]:

(2) Y=0.299R+0.587G+0.114B,

Figure 5 below presents the calculation results graphically. The horizontal axis shows the numbers of the successive test fields, while the vertical axes display the color medians and the mean brightness according to Equation (1), as well as the mean pixel luminance according to Equation (2). The numbers of the test panels (fields) have been assigned randomly and result solely from the scanning sequence.

As shown in Figure 5, the color range of the measurement panels is not highly diverse. The lowest values of the color components correspond to blue, while the highest correspond to red. Different pigments in the panels have different light absorption characteristics. Due to that, Figure 5 shows a systematic distribution of colors (the blue color has a lower average, while the red color has a higher average). The pigments in the blue-colored panels have greater light absorption, which leads to a weaker return signal and lower component values in the scans. On the other hand, red pigments have a higher light reflection coefficient, which results in higher values.

Analyzing the charts for luminance and mean brightness, similar values were obtained for all measured control panels. The panel colors can be considered bright, as all luminance and mean brightness values exceed 50% of the maximum value.

## 5. Analysis of Measurement Data

**Figure 6:**
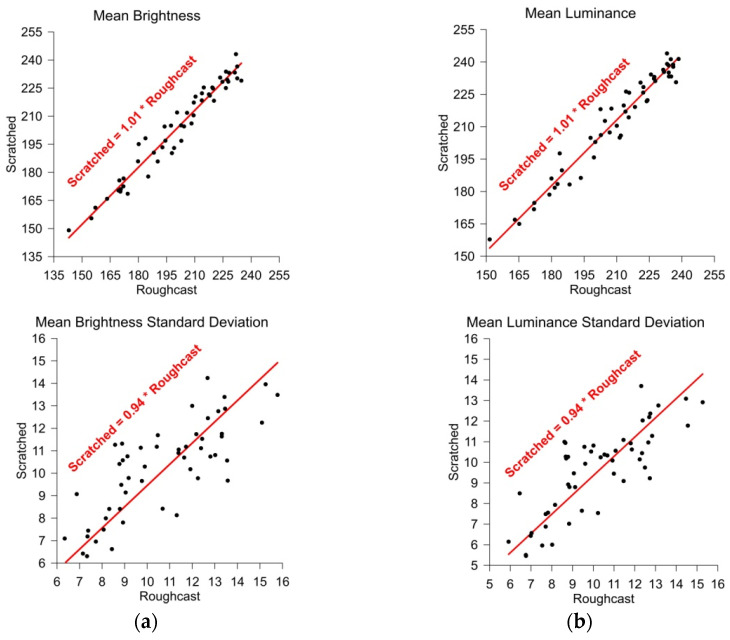
Comparison of the average values of mean brightness and luminance between renders of the same color: (**a**) mean brightness; and (**b**) mean luminance.

In order to determine whether different renders on the same panel differ in brightness parameters, the average values of mean brightness and mean luminance were compared, along with their respective averages for both types of render. The results of these analyses are presented below in Figure 6. The horizontal axis contains data corresponding to the Roughcast-type renders, while the vertical axis of the charts shows data corresponding to Scratched-type ones. An analysis of the relation between variables was conducted using the Pearson correlation.

(3) $$r=\frac{\sum_{i=1}^{n}\left(x_{i}-\bar{x}\right)\cdot \left(y_{i}-\bar{y}\right)}{\sqrt{\sum_{i=1}^{n}{\left(x_{i}-\bar{x}\right)}^{2}\cdot \sum_{i=1}^{n}{\left(y_{i}-\bar{y}\right)}^{2}}},$$

where

$x_{i},y_{i}$—two variables
$\bar{x},\bar{y}$—mean of variables

The data in Table 1 indicates that there are very strong positive correlations between the analyzed variables.

Black dots in Figure 6 represent individual panels, while in red color there are presented fitted lines. Different variants of approximation lines were tested, and it was ultimately decided that “Through origin” functions allow us to describe the measurement points best.

When comparing the results shown in Figure 6, it can be concluded that for both mean brightness (Figure 6a) and mean luminance (Figure 6b), the values obtained for both types of render follow linear “Through origin” functions. This indicates that it can be assumed that the render colors on each test panel were the same. Minor differences may result from measurement accuracy and data dispersion. The distances of the plotted points from the approximated line are small, which confirms a good color match between different types of render of the same color (on the same test panel). Figure 6 also presents the standard deviations of mean brightness and mean luminance for each test field. In this case, greater differences in the results can be observed. The theoretical curve (red line) indicates that the random dispersion of Roughcast-type renders is slightly higher—by about 6%—than that of Scratched-type ones. This value was obtained with considerable dispersion (large distances from the fitted line), so while the result is interesting, it can be considered negligible for the purposes of further analyses.

The laser scanning results, in the form of point clouds, are in the scanner’s internal coordinate system. To perform analyses between individual control panels, the point clouds were transformed into a coordinate system associated with the measurement frame (the geometric center of each cloud) in the *XZ* plane, while the *Y* coordinate is perpendicular to the plane of the measurement frame. As a result of the transformation, square grids of measurement points were obtained, with dimensions listed below in Table 2.

As shown in Table 2, after the transformation, all points were brought into a single 3D coordinate system. The maximum distance between the scanner and the test panels recorded during the measurements was 8.0221 m, and the minimum was 8.0029 m, meaning the greatest discrepancy in scanner-to-panel distance measurements was 0.0192 m.

The first step was to analyze the results between the types of render on each of the *m* = 52 test panels, where two fields of *n* = 4489 points each were measured. The following statistical parameters were determined based on the measurements:

- The distance between the extreme values of the measured distances within the test field *k*:
  (4) $$\;dY_{k}=\underset{j=1,2,\ldots ,n}{\max}Y_{k,j}-\underset{j=1,2,\ldots ,n}{\min}Y_{k,j}.$$
- The mean value of the measured distances within the test field *k*:
  (5) $$\;{\bar{Y}}_{k}=\frac{1}{n}\sum_{j=1}^{n}Y_{k,j}.$$
- The standard deviation of the measured distances within the test field *k*:
  (6) $$\forall j=1,2,\ldots n,\;\mathrm{StDev}\left(Y_{k}\right)=\sqrt{\frac{1}{n-1}\sum_{j=1}^{n}{\left(Y_{k,j}-{\bar{Y}}_{k}\right)}^{2}}.$$

**Figure 7:**
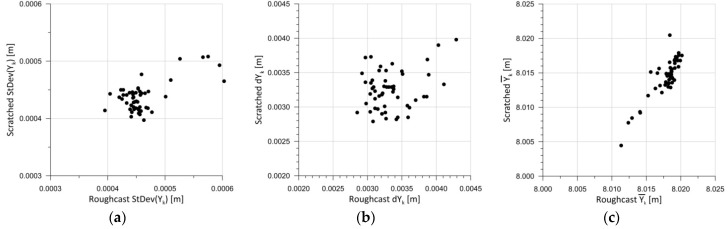
Comparison of parameters of measurement distances for different renders: (**a**) standard deviation; (**b**) distance between max and min distance; and (**c**) mean distance within the test field.

Figure 7 presents parameters (4)–(6) for each test panel. The horizontal axis shows the results for Roughcast-type render, while the vertical axis displays the results for Scratched-type one. This graphical presentation of the results enables a direct comparison between two render types from the same panel.

**Figure 8:**
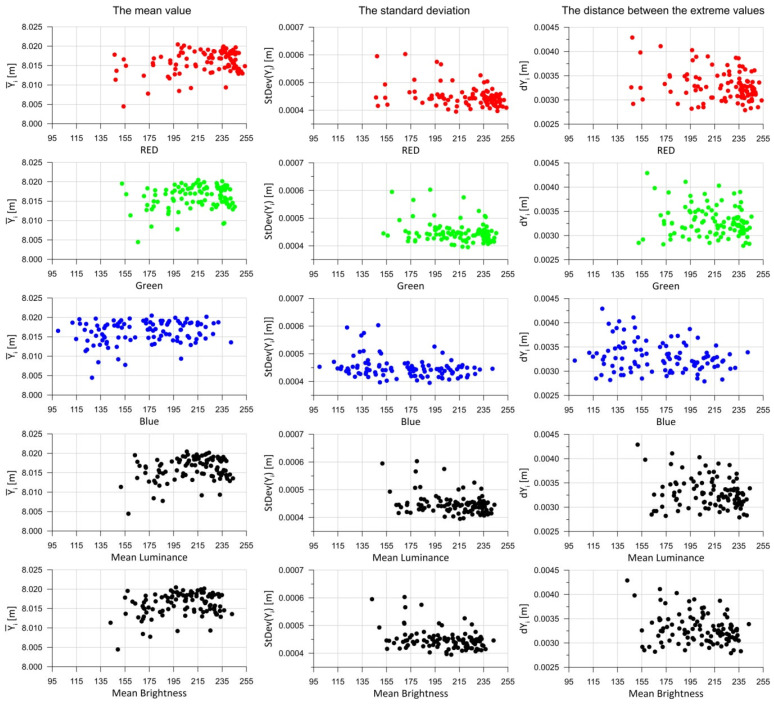
The mean distance, standard deviation, and measured distance range analyzed as functions of RGB, mean brightness, and mean luminance.

When analyzing the statistical parameters of the test panel measurements presented in Figure 7, it can be concluded that the standard deviation values are very low for the entire set of panels (Figure 7a). In practice, the standard deviation of the measurements is around 0.5 mm. This is a result of scanning points at 3 mm intervals, yielding 4489 points per test field. With such a high number of points, the standard deviation remains low. The distribution of discrepancies between the maximum and minimum distance measurements within a test field (Figure 7b) is far more interesting. These discrepancies range between 2.8 mm and 4.3 mm. It was assumed that abovementioned differences resulted mainly from the measurement accuracy of TLS during the experiment. Figure 7c shows the relationship between the mean distance values within the test field for each test panel. In this case, a significant linear correlation between the obtained mean values can be observed, which is also a satisfactory result. However, no significant dependency is visible between the test fields on the same panel. A linear relationship might be inferred, but such a result would carry a high approximation error. Further analysis regarding the color components and brightness of the test fields was conducted collectively, meaning for all *m* = 104 fields. Statistical parameters were calculated according to Formulas (4)–(6). Figure 8 below presents 15 charts. The first column shows the results for the mean distances within the test fields (5). The second column contains the standard deviations for each test field (6), while the third column presents the extreme measured distances (4).

In relation to the RGB color components, it is difficult to identify any functional dependencies between the colors and the analyzed statistical parameters (Figure 8). However, it can be observed that as the values of the color components, mean luminance, and mean brightness increase, the dispersion of the statistical parameter values decreases (the results become more consistent—the point cloud narrows). With regard to mean luminance and mean brightness, some functional relationships may also be inferred, but the dispersion of points on the charts is significant, making any approximation result highly imprecise (many values fall outside the regression).

Further analysis was conducted on measurements between different control panels. To perform this analysis, the first step involved recalculating the measurement points on each test field to a uniform point grid with a mesh size of 3 × 3 mm. This resulted in a consistent set of points (*n* = 4489), with 67 columns and 67 rows in each test field. The distance interpolation was performed using the Inverse Distance to Power method [63]. The points were then analyzed between test fields, separately for Roughcast-type and Scratched-type renders (*m* = 52). For each point, the mean distance value was calculated according to Formula (5), as well as the standard deviation:

(7) $$\forall j=1,2,\ldots n,\forall k=1,2,\ldots ,m,\;\mathrm{StDev}2\left(Y_{j}\right)=\sqrt{\frac{1}{nm-1}\sum_{k=1}^{m}{\left(Y_{k,j}-{\bar{Y}}_{j}\right)}^{2}},$$

**Figure 9:**
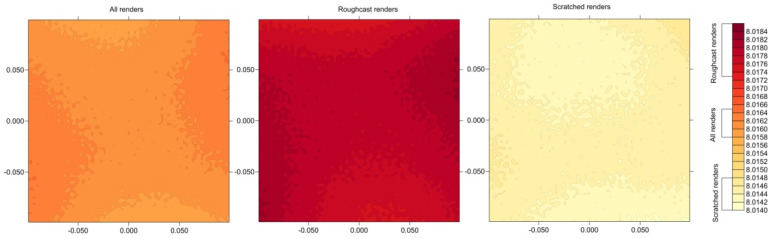
Mean distance in analysis.

Below, Figure 9 presents the distributions of mean distances to a given point on the test field for all renders, as well as separately for Roughcast-type and Scratched-type renders. As shown, the maximum discrepancies in mean distances to a given point are approximately 1 mm. As a result, there might be an attempt to identify a functional distribution of distances, but given the very small differences in mean distances between points on the control panel, such an analysis is not justified.

**Figure 10:**
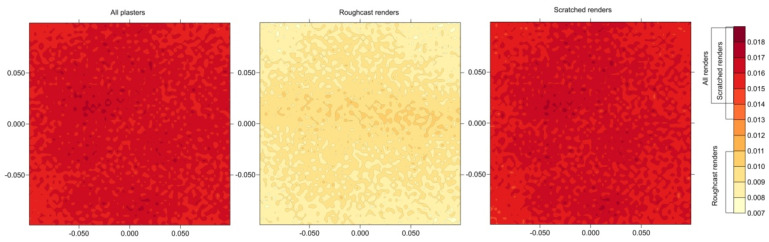
Range *dY* in points analysis.

The analysis was also conducted on the distribution of result discrepancies at a given point, denoted as *dY* (the difference between the maximum and minimum value). Figure 10 presents the *dY* discrepancies in the form of color maps. It should be noted that smaller discrepancies were recorded for Roughcast-type renders, ranging from 7 to 11 mm, while for Scratched-type ones, the discrepancies ranged from 13 to 19 mm. For the cumulative results of all renders, the calculations for Scratched renders had the greatest significance.

**Figure 11:**
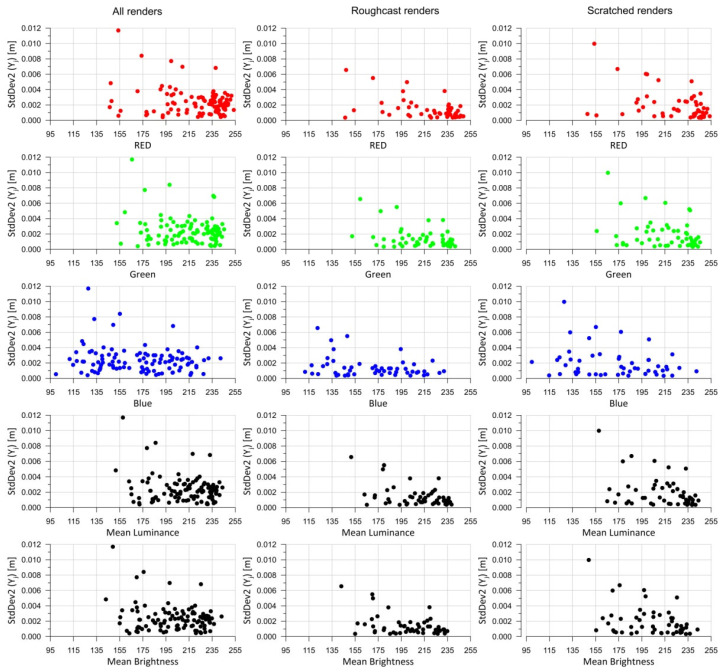
Analyze of standard deviation (6) in points for all renders and separately for Roughcast-type renders and Scratched-type ones.

Based on the mean distances measured to a given point on the measurement grid, standard deviations (*StDev2*) were calculated in accordance with Equation (7). The obtained results are presented graphically in Figure 11, showing the function for all panels as well as for individual render types, depending on their color components, mean brightness, and mean luminance.

The results of the standard deviation calculations shown in Figure 11—comparing the mean values at specific points with those measured on individual test fields—do not reveal significant differences that would allow for a clear interpretation. In the graphs related to mean brightness and mean luminance, it can be observed that as the brightness increases, the standard deviation *StdDev2* (7) decreases. However, due to the fact that the result clouds are broad, the approximation using a theoretical function does not yield reliable results. Interestingly, as the mean brightness and mean luminance decrease, the dispersion of results obtained increases for different test fields but with similar brightness parameters. Regarding the RGB color components, the patterns for the green and red components resemble those of brightness and luminance. In contrast, the results for the blue component are more scattered, with the result cloud appearing almost parallel to the horizontal axis.

**Figure 12:**
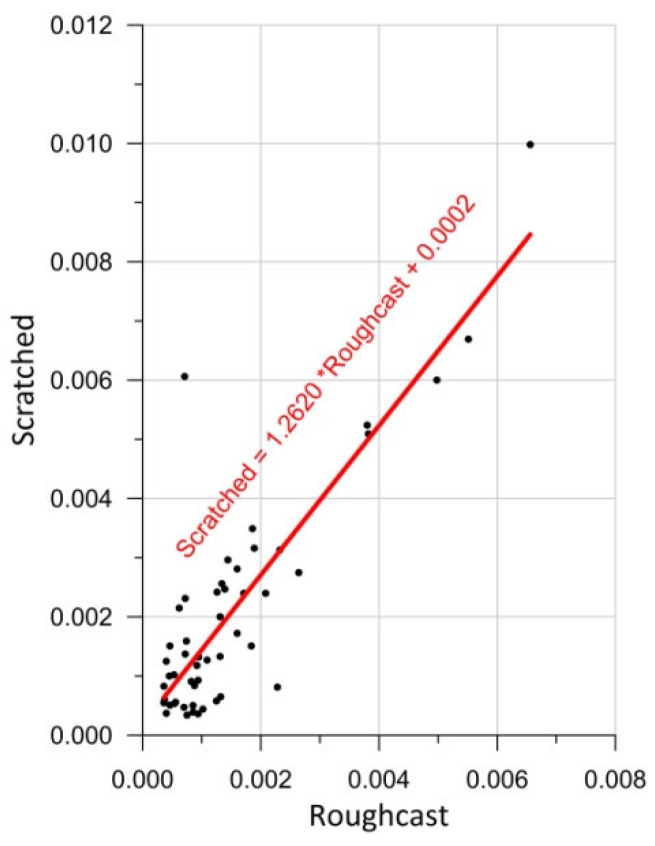
Comparison between StDev2 (6) of Roughcast-type and Scratched-type renders.

Subsequently, the analysis was conducted on the standard deviation values (*StDev2*) depending on the types of render. Since, according to Figure 6, different renders on a given panel share the same color parameters, it was decided to compare the renders in pairs. The results of this analysis are presented in Figure 12.

Pearson’s correlation (3) was used to check the relationship between variables. The Pearson correlation *r* was calculated at 0.857, which confirms a strong linear correlation between the standard deviations for both types of render. Therefore, it was decided to approximate the results using a linear function. According to this approximating function, Scratched-type renders exhibit an approximately 26% higher standard deviation compared to the Roughcast-type ones.

## 6. Conclusions

The research presented in this paper aimed to assess the impact of decorative render parameters—in particular their type, color, and brightness—on the quality and accuracy of measurements performed using terrestrial laser scanning (TLS). Based on the conducted laboratory experiment, a number of conclusions were drawn, the most important of which are

- The surface texture affects the precision of the TLS measurements.
- The analysis showed that renders with the Scratched-type texture generate an approximately 26% higher standard deviation of distance measurements compared to the Roughcast-type ones.
- The surface brightness influences the consistency of measurement results.
- The clear trend was observed that the dispersion of the TLS statistical parameters increases as the render brightness decreases.
- Darker surfaces reduce laser light reflectivity, resulting in decreased data repeatability.
- The influence of RGB color components on measurement results proved to be minor, with blue-colored renders causing the smallest deviations, suggesting that this color interferes the least with a laser light reflection.

The abovementioned conclusions are subject to limitations due to the restricted range of comparative materials in terms of their brightness and color. Although the study conducted on 104 test fields provided a substantial amount of data, future research could explore a broader range of renders colors and textures. The study confirmed that the texture and brightness of render surfaces are significant factors when modeling objects based on TLS data. In applications requiring high surface modeling precision—such as architectural diagnostics or conservation works—the type and optical properties of materials should be taken into account. Choosing brighter and less textured surfaces may contribute to improved measurement quality.

The designed research methodology proved effective (in analyses of the surface structure of the test plates). The use of a custom-built frame for positioning the panels, uniform lighting conditions, and surface brightness analysis based on high-resolution scans enabled the acquisition of repeatable and reliable comparative results.

The study confirms that despite its high precision, the TLS technology can be sensitive to the microtexture and optical properties of scanned materials, which should be considered when planning measurements and interpreting results (for example, in studies of deformation of tunnel linings).

## Figures and Tables

**Table 1:** The results of analysis of the relation between renders with the Pearson correlation “r”.

	Brightness	Luminance
Mean	0.975	0.975
Standard Deviation	0.787	0.809

**Table 2:** Statistical data from 104 grids.

	Min	Max	Mean
x	−0.0997	0.0997	0.0000
y	8.0029	8.0221	8.0161
z	−0.0999	0.0996	0.0000

## Data Availability

All research data are available via email contact.
